# Factors affecting complete and timely childhood immunization coverage in Sindh, Pakistan; A secondary analysis of cross-sectional survey data

**DOI:** 10.1371/journal.pone.0206766

**Published:** 2018-10-31

**Authors:** Jin-Won Noh, Young-mi Kim, Nabeel Akram, Ki-Bong Yoo, Jumin Park, Jooyoung Cheon, Young Dae Kwon, Jelle Stekelenburg

**Affiliations:** 1 Department of Healthcare Management, Eulji University, Seongnam, Korea; 2 Global Health Unit, Department of Health Sciences, University Medical Centre Groningen, University of Groningen, Groningen, the Netherlands; 3 Jhpiego, Johns Hopkins University, Baltimore, Maryland, United States of America; 4 Department of Health Administration, College of Health Sciences, Yonsei University, Wonju, Korea; 5 National Institutes of Health Clinical Center, Bethesda, Maryland, United States of America; 6 Department of Nursing Science, Sungshin University, Seoul, Korea; 7 Department of Humanities and Social Medicine, College of Medicine and Catholic Institute for Healthcare Management, The Catholic University of Korea, Seoul, Korea; 8 Department of Obstetrics and Gynecology, Medical Centre Leeuwarden, Leeuwarden, the Netherlands; University of Campania, ITALY

## Abstract

**Background:**

Pakistan has a high burden of newborn mortality, which would be significantly preventable through appropriate routine immunization. The purpose of this study was to measure the basic timely childhood immunization coverage and to identify determinants of factors influencing childhood immunization coverage in Sindh, Pakistan.

**Methods:**

Data from Maternal and Child Health Program Indicator Survey 2013–2014 which was conducted in Sindh province of Pakistan was used. Outcome measure was full coverage of the basic immunization schedule from child’s vaccination card. The association of receiving basic immunization with demographic factors, socioeconomic status, mother and child health information sources, and perinatal care factors were tested by binary logistic regression.

**Results:**

Among 2,253 children, 1,156 (51.3%) received age-based full basic immunization. The basic immunization rates were 69.1% for under five weeks old, 38.3% for six to nine weeks, 18.8% for 10–13 weeks, 44.0% for 14 weeks-eight months, 60.4% for nine to 11 months, and 59.1% for over one year. Child’s age, number of living children, parents’ education level, wealth, the source of mother and child health information, number of antenatal care, and assistance during delivery were associated with completing basic immunization.

**Conclusions:**

The overall full basic immunization coverage in Pakistan was still low. Policy makers should identify children at risk of low immunization coverage and obstacles of receiving antenatal care, implement educational interventions targeting on less educated parents, and conduct mass immunization campaigns for timely and complete immunization.

## Introduction

Children across Pakistan are at risk of falling ill with life-threatening diseases because of lack of access to vaccinations. Approximately, 400,000 children under five years of age die every year from vaccine-preventable diseases in Pakistan [1]. The Pakistan Demographic Health Survey from 2013 estimated that almost three million children dropped out of the basic course of vaccines every year [1]. The survey of Pakistan Social and Living Standards Measurement (2014–15) showed that Pakistan’s full immunization coverage was 60%. In addition, the coverage rate stood at 27–70% with large variations between the provinces, districts and communities [2].

Given the low vaccination rates in Pakistan, immunization programs play an essential role in the strategy to raise the level of herd immunity, ultimately reducing child mortality. In communities with herd immunity through sufficient immunization coverage of the population, vulnerable children are protected because the majority of individuals they come into contact with are immune and therefore incapable of spreading communicable disease [3]. In addition, timing of childhood immunization is critical because if children are immunized too early or if the immunizations are too closely spaced, it can significantly shorten the duration of protection or interfere with the immune response [4, 5]. Delayed immunizations lead to prolonged potential exposure to vaccine-preventable diseases [6, 7]. Hence, it is important to assess when, not just if, children are receiving immunizations. The current immunization schedule in Pakistan was first introduced in July 2011 [8] (Table 1).

**Table 1:** Immunization schedule in Pakistan and definition of full basic immunization.

Immunization schedule		Definition of full basic immunization, by age group	
Age	Immunizations	Age group	Requirements for full basic immunization
At birth	BCG+ Polio 0	0–5 weeks	BCG, Polio 0
6 weeks	Penta 1 + Polio 1	6–9 weeks	BCG, Polio 0, Polio 1, Penta 1
10 weeks	Penta 2 + Polio 2	10–13 weeks	BCG, Polio 0, Polio 1, Polio 2, Penta 1, Penta 2
14 weeks	Penta 3 + Polio 3	14 weeks– 8 months	BCG, Polio 0, Polio 1, Polio 2, Polio 3, Penta 1, Penta 2, Penta 3
9 months	Measles 1	9–11 months	BCG, Polio 0, Polio 1, Polio 2, Polio 3, Penta 1, Penta 2, Penta 3, Measles 1
12–15 months	Measles 2	12 months– 23 months	BCG, Polio 0, Polio 1, Polio 2, Polio 3, Penta 1, Penta 2, Penta 3, Measles 1, Measles 2

BCG: Bacillus Calmette–Guérin (BCG) is an antituberculosis vaccine.

Penta: Pentavalent vaccine for diphtheria, tetanus, acellular pertussis, poliomyelitis, and Haemophilus influenzae type b

Understanding factors that influence immunization coverage is essential to increase routine immunization coverage rates. Several studies found that substantial health inequity according to socioeconomic status, such as residency [9], wealth [9–11], educational status [9, 11, 12], and number of children in a household [13, 14], affects immunization coverage. In addition, immunization coverage is influenced by mother and child health information sources, such as antenatal care (ANC) visits [15] and accessibility to mass media [10, 11, 16].

Information about factors that influence immunization coverage might be valuable for healthcare providers and policy makers to develop and provide effective programs, contributing to the increase in the childhood immunization coverage rates [1]. Although the need to improve routine immunization across all regions and districts in Pakistan has been strongly emphasized [1], influencing factors have not been examined in depth. The purpose of this study was to measure the basic timely childhood immunization coverage and to identify determinants such as age group, socio-demographic characteristics, information sources, and perinatal care that influence childhood immunization coverage in Sindh, one of the four provinces of Pakistan.

## Methods

### Data and subjects

We used survey data in 2013 and 2014 from the Maternal and Child Health (MCH) Program Indicator Survey which was conducted in Pakistani province of Sindh [17]. Sindh province is located in the southeastern part of Pakistan. Its area is 140,914 km^2^ and it includes approximately 44 million people [18]. In Sindh, total fertility rate was 3.9, the under-five mortality rate was 93 deaths per 1,000 live births, and the infant mortality rate was 74 per 1,000 live births from the results of Pakistan Demographic and Health Survey (PDHS) 2012–2013 [18].

The MCH Program Indicator Survey was set to monitor the implementation of maternal, newborn, and child health (MNCH) and family planning/reproductive health interventions by the United States Agency for International Development (USAID) [17]. The survey instrument was based on the PDHS instrument developed by Macro International, Inc. and the Knowledge, Practice and Coverage Survey instrument developed by the Johns Hopkins University/Child Survival Support Program 1990 [17]. The study team pilot tested the survey questionnaire in the local languages of Urdu and Sindhi.

This survey data is from 23 districts of Sindh province. The sample is representative of urban and rural areas of Sindh. It is a cross-sectional study using a multi-stage, stratified sampling design. Based on the most recent Census of Pakistan in 1998, USAID used a disproportionate sampling approach to allocate the sample in districts in rural and urban areas for better representation of smaller districts. Then probability proportionate to size method was used to select cities and villages. USAID allocated a maximum of 10 participants in each village and 15–200 participants in each selected city to take part in the study. Finally, data were collected in all 23 districts of Sindh from June to October in 2013 and 2014 [17]. The study participants included married women 15–49 who had a live birth in the two years prior to the survey and who resided in the houses sampled for study participation. Only one study participant was selected from a household. Each woman completed questionnaires about her last live birth. An assessment of data quality was conducted by the director of Monitoring and Evaluation of the MNCH Services Component after the data were made available for analysis. Both internal and external validity checks were conducted.

The study was approved by the Johns Hopkins University School of Public Health Institutional Review Board (IRB00005002) and the National Bioethics Committee of Pakistan. Both Institutional Review Boards approved the verbal consent. Female interviewers obtained informed consent verbally from each respondent and then signed the consent form on behalf of the respondent. Interviewers were required to sign the consent form attached to the survey questionnaire to confirm that they had read the informed consent script.

### Outcome measure

The primary outcome variable was full coverage of the basic immunization schedule (Table 1). Survey respondents were asked to show their child’s vaccination card, so that data collectors could collect their immunization records. Secondly, we evaluated the percent of children in a certain age group who had received all of the immunizations as per the national recommended immunization schedule for a child that age. For this purpose, we categorized children’s age into under five weeks, six to nine weeks, 10 weeks-13 weeks, 14 weeks–eight months, nine months-11 months, and 12 months-23 months. Definitions of full basic immunization per age group are presented in Table 1.

### Independent variables

Demographic factors, socioeconomic status, MCH information sources, and factors regarding perinatal care were included to identify the factors associated with immunization conditions in this study. These independent variables were derived from previous studies [19, 20] and MCH Program Indicator Survey report [17].

Mother’s age and number of living children were included as demographic factors. The mother’s age was classified into 15–24, 25–34, 35 and older, and number of living children was classified into one, two, three, four, and five- based on MCH Program Indicator Survey report [17]. To determine socioeconomic status of the respondents, data was collected on rural-urban residence, mother’s and father’s education, and household wealth. Wealth index was calculated by principal components analysis based on the household ownership of assets [8]. Principal component analysis is a well-known statistical method to reduce dimensionality [21] and was used to assess household wealth based on the value of 35 households’ assets. Thus, we made a wealth variable from the value of 35 household assets. After calculating the index, it was classified into quintiles.

MCH information source was assessed by asking, “During the last 12 months have you received any information about MCH from the following sources?” Responses were categorized into health professionals (doctor, nurse/midwife, lady health visitor), lower-level health workers (Dai-traditional birth attendant, lady health worker, homeopath, Hakim-herbal medicine practitioner, outreach worker), relatives/friends, and media (radio, TV, telephone helpline, text message on mobile phone, health education/awareness session, print media). Binary variables (yes/no) for each response in MCH information source were included. Number of ANC visits, assistance during delivery, and place of delivery were included as factors of perinatal care.

### Statistical analysis

Data from 2013 and 2014 were pooled for this analysis. A chi-squared test was used to test distribution of general characteristics for the bivariate analysis, between full basic immunization and independent variables. For multivariate analysis, binary logistic regression was used to investigate factors associated with full basic immunization. The criterion for significance was p≤0.05, two-tailed. Odds ratio (OR) and 95% confidence interval (CI) were calculated. We presented the crude OR and adjusted OR in our results table. The adjusted OR were results of binary logistic regression by adjusting all the independent variables (children’s age, mother’s age, number of living children, residence, mother’s education, father’s education, wealth, MCH information source, number of ANC visits, assistance during delivery, place of delivery). All analyses were performed in SAS version 9.4 **(**SAS Institute, Cary, NC, USA).

## Results

**Fig 1:**
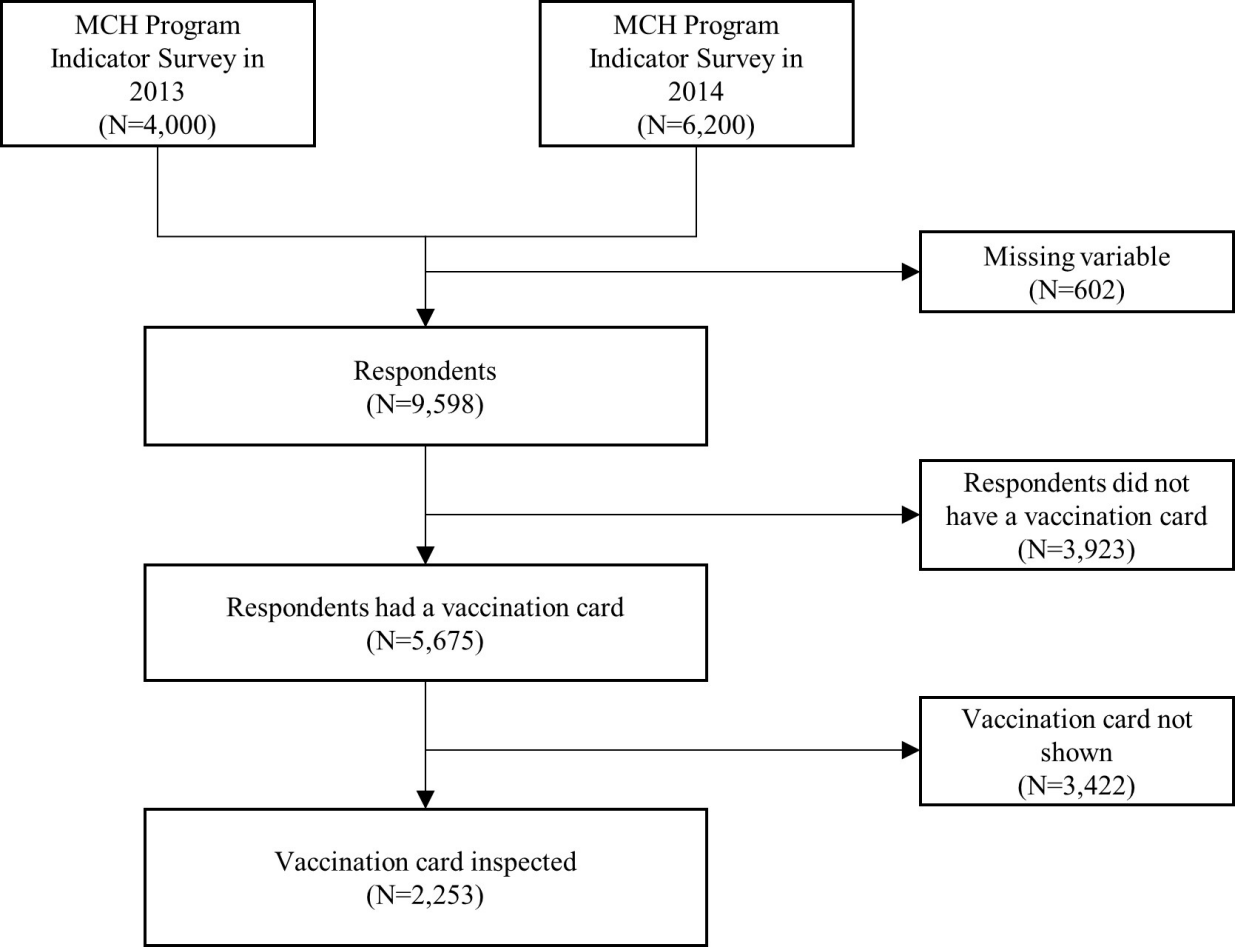
Flow chart showing study population selection.

Data were collected from a total of 10,200 women, 4,000 women in 2013 and 6,200 women in 2014. Details of the study population selection is shown in Fig 1; 602 subjects were excluded because of missing variables for wealth (N = 34), father’s education level (N = 94), mother’s education level (N = 21), mean number of living children (N = 23), ANC use information (N = 309), and immunization care (N = 121) (Fig 1). Then respondents who did not have a vaccination card (N = 3,923) and did not/could not show a vaccination card (N = 3,422) were excluded. Finally, 2,253 subjects were selected as study population.

**Fig 2:**
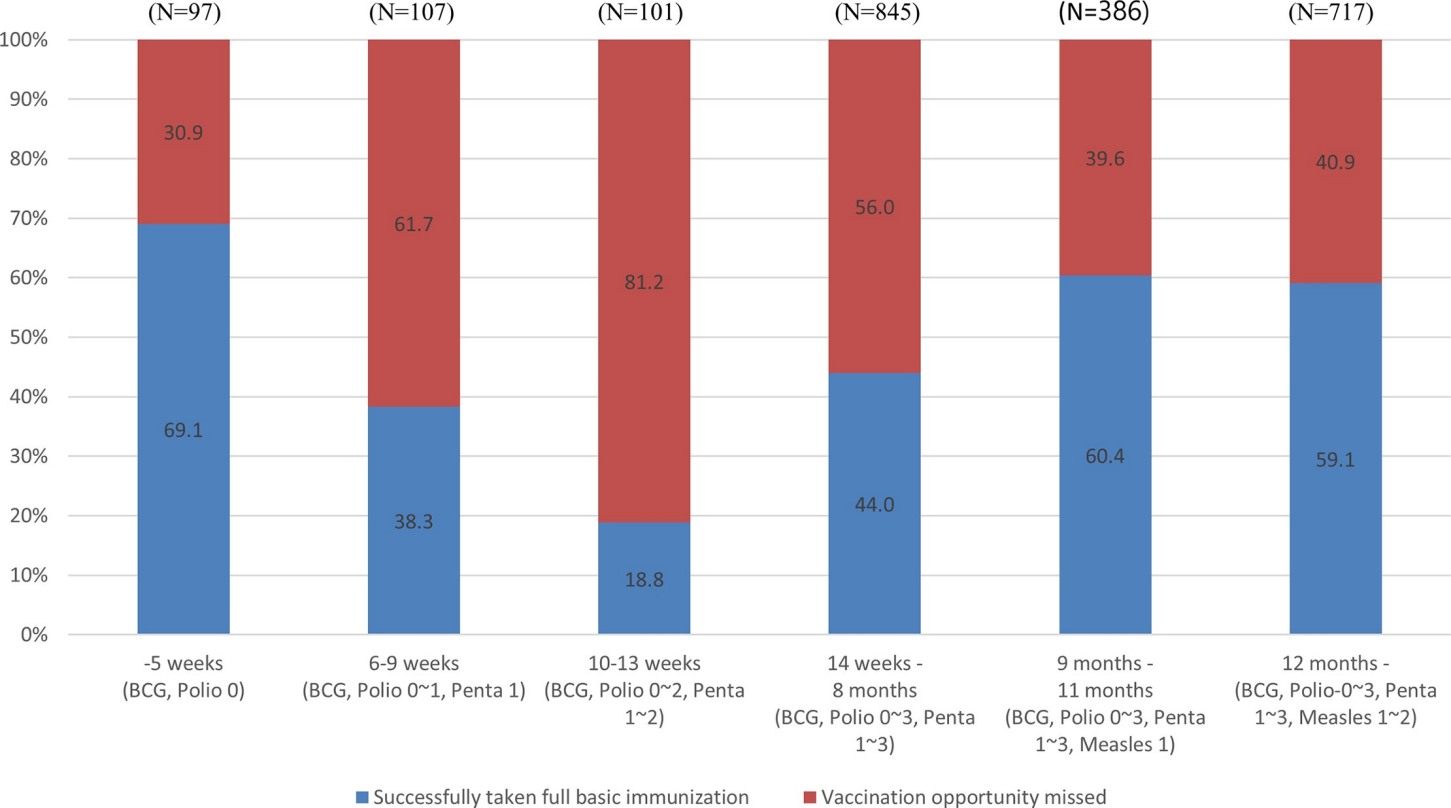
Proportion of children vaccinated on schedule between 2013 and 2014.

Table 2 shows the general characteristics of the mothers and children that were included in the study. Among 2,253 children, 1,156 children (51.3%) received age-based full basic immunization. The percentage of children having received full basic immunization varied by children’s age (Fig 2); 69.1% of the children under five week old had received full basic immunization, compared with 38.3% for children aged six weeks—nine weeks and just 18.8% for children age 10 weeks-13 weeks.

**Table 2:** Percentage of children age 0–23 months who have completed full basic immunization, by age group, sociodemographic characteristics, information sources, and perinatal care.

		Full basic immunization		Total (n = 2,253)	p-value
		Yes (n = 1,156)			
		N	%	N	
Children’s age	0–5 weeks	67	69.1	97	< .001
	6–9 weeks	41	38.3	107	
	10–13 weeks	19	18.8	101	
	14 weeks-8 months	372	44.0	845	
	9–11 months	233	60.4	386	
	12–23 months	424	59.1	717	
*Demographic factors*					
Mother's age	15–24	381	49.0	777	0.287
	25–34	652	52.6	1239	
	35-	123	51.9	114	
Number of living children	1	315	47.6	662	0.083
	2	308	55.3	557	
	3	193	52.2	370	
	4	136	53.5	254	
	5+	204	49.8	410	
*Socioeconomic status*					
Residence	Rural	331	47.8	692	0.089
	Town/Small city	420	53.0	793	
	Large city	405	52.7	768	
Mother’s Education	No education	420	44.5	943	< .001
	Primary or middle	328	52.6	624	
	Secondary or higher	408	59.5	686	
Father's education	No education	248	43.1	576	< .001
	Primary or middle	251	48.5	517	
	Secondary or higher	657	56.6	1,160	
Wealth quintile	First (poorest)	60	35.1	171	< .001
	Second	124	44.4	279	
	Third	250	52.2	479	
	Fourth	315	49.7	634	
	Fifth (richest)	407	59.0	690	
*Information about maternal and child health received from*:					
Health professional	No	486	44.9	1,083	< .001
	Yes	670	57.3	1,170	
Low-level health workers^†^	No	1027	51.5	1993	0.561
	Yes	129	49.6	260	
Relatives/friends	No	476	44.7	1064	< .001
	Yes	680	57.2	1189	
Media^‡^	No	734	47.3	1,551	< .001
	Yes	422	60.1	702	
*Health care during pregnancy and delivery*					
Number of antenatal care visits	1–2	160	40.9	391	< .001
	3	167	53.7	311	
	4+	829	53.4	1,551	
Assistance during delivery	Traditional birth attendant	945	53.1	1,779	< .001
	Health professional	205	45.7	449	
	No one/others	6	24.0	25	
Place of delivery	Home	239	44.1	542	< .001
	Private facility	718	54.1	1,327	
	Public facility	199	51.8	384	

†Low-level health workers included Dai-traditional birth attendant, lady health worker, homeopath, Hakim-herbal medicine practitioner, and outreach worker.

‡Media included radio, TV, telephone helpline, print media, health education/awareness session, and text message on mobile phone.

Based on the results of chi-square test, children’s age, mother’s education, father’s education, wealth quintile, MCH information source (health professional, relatives/friends, media), number of ANC visits, assistance during delivery, and place of delivery were significant. The proportion steadily increased among older age groups, from 44.0% of children age 14 weeks-eight months, to 60.4% of children aged nine-11 months, and 59.1% of children aged one year. Higher education level in both father and mother showed higher proportion of full basic immunization. High wealth group showed a high proportion of full basic immunization, but the proportion of the fourth wealth group (49.7%) was lower than the third wealth group (52.2%). Women who received MCH information from health professionals, relatives/friends, and media showed a higher proportion of full basic immunization than those who did not. Women who only visited the ANC clinic 1–2 times showed the lowest proportion of full basic immunization (40.9%). The proportion of full basic immunization in women whose birth was assisted by a traditional birth attendant was 53.1%. It was 45.7% in health professional, 24.0% in no one/others. Women who delivered at home showed the lowest proportion (44.1%), compared to 54,1% in women who gave birth in private facilities, and 51,8% in women who gave birth at public facilities (Table 2).

Table 3 shows the results of crude and adjusted binary logistic regression. Compared to children who were under five weeks old, older children were less likely to have completed age-based basic immunization except children who were nine-11 months. Children who were nine-11 months showed OR = 0.64, but it was not significant compared to children who were under five weeks old. Hence, the proportion of children with completed full basic immunization was higher for children age under five weeks. Compared to the children under five weeks old, the OR of children who were six-nine weeks were 0.25 (95% CI, 0.14–0.45), the OR of children who were 10–13 weeks old showed the lowest result (OR, 0.09; 95% CI, 0.05–0.18), the OR of children who were 14 weeks–eight months were 0.34 (95% CI, 0.21–0.54) and the OR of children who were over 12 months were 0.59 (95% CI, 0.37–0.94) (Table 3).

**Table 3:** Results of logistic regression for full basic immunization (n = 2,253).

		Crude			Adjusted^†^		
		OR	95% Confidence Interval		OR	95% Confidence Interval	
Children’s age	0–5 weeks	1.00			1.00		
	6–9 weeks	0.28	0.16	0.50	0.25	0.14	0.45
	10–13 weeks	0.10	0.05	0.20	0.09	0.05	0.18
	14 weeks-8 months	0.35	0.22	0.55	0.34	0.21	0.54
	9–11 months	0.68	0.42	1.10	0.64	0.39	1.05
	12–23 months	0.65	0.41	1.02	0.59	0.37	0.94
*Demographic factors*							
Mother's age	15–24	1.00			1.00		
	25–34	1.15	0.97	1.38	1.00	0.81	1.23
	35-	1.12	1.84	1.50	0.98	0.69	1.40
Number of living children	1	1.00			1.00		
	2	1.36	1.09	1.71	1.40	1.10	1.78
	3	1.20	0.93	1.55	1.24	0.94	1.65
	4	1.27	0.95	1.70	1.47	1.06	2.03
	5+	1.09	0.85	1.40	1.42	1.04	1.93
*Socioeconomic status*							
Residence	Rural	1.00			1.00		
	Town/small city	1.23	1.00	1.51	0.96	0.75	1.23
	Large city	1.22	0.99	1.50	0.78	0.59	1.04
Mother’s education	No education	1.00			1.00		
	Primary or middle	1.38	1.13	1.69	1.17	0.93	1.49
	Secondary or higher	1.83	1.50	2.23	1.37	1.04	1.80
Father's education	No education	1.00			1.00		
	Primary or middle	1.25	0.98	1.58	1.08	0.83	1.40
	Secondary or higher	1.73	1.41	2.11	1.30	1.03	1.65
Wealth	First (poorest)	1.00			1.00		
	Second	1.48	0.99	2.19	1.25	0.83	1.90
	Third	2.02	1.41	2.90	1.52	1.01	2.27
	Fourth	1.83	1.29	2.59	1.21	0.79	1.88
	Fifth (richest)	2.66	1.88	3.77	1.53	0.95	2.45
*Information about maternal and child health received from*:							
Health professional	No	1.00			1.00		
	Yes	1.65	1.39	1.94	1.24	1.02	1.52
Low-level health workers^‡^	No	1.00			1.00		
	Yes	0.93	0.72	1.20	1.06	0.79	1.44
Relatives/friends	No	1.00			1.00		
	Yes	1.65	1.40	1.95	1.38	1.12	1.71
Media^¶^	No	1.00			1.00		
	Yes	1.68	1.40	2.01	1.32	1.08	1.61
*Health care during pregnancy and delivery*							
Number of antenatal care visits	1–2	1.00			1.00		
	3	1.67	1.24	2.26	1.61	1.17	2.22
	4+	1.66	1.32	2.08	1.39	1.09	1.79
Assistance during delivery	Traditional birth attendant	1.00			1.00		
	Health professional	1.35	1.10	1.66	0.72	0.45	1.14
	No one/others	0.38	0.15	0.96	0.26	0.10	0.71
Place of delivery	Home	1.00			1.00		
	Private facility	1.50	1.22	1.83	1.54	0.99	2.39
	Public facility	1.36	1.05	1.77	1.50	0.94	2.41

†Children’s age, mother’s age, number of living children, residence, mother’s education, father’s education, wealth, maternal and child health information source, number of antenatal care use, assistance during delivery, and place of delivery were adjusted.

‡Low-level health workers included Dai-traditional birth attendant, lady health worker, homeopath, Hakim-herbal medicine practitioner, and outreach worker.

¶Media included radio, TV, telephone helpline, print media, health education/awareness session, and text message on mobile phone

Notes. OR>1: successfully taken full basic immunization

Living with two children (OR, 1.40; 95% CI, 1.10–1.78) or four children (OR, 1.47; 95% CI, 1.06–2.03) or five and more children (OR, 1.42; 95% CI, 1.04–1.93) did increase likelihood of full basic immunization compare to only one child. Parents’ education level, which was secondary or higher group, was significantly and positively associated with completing basic immunization. Mothers who were educated at the level of secondary or higher showed significant odds ratios (OR, 1.37; 95% CI, 1.04–1.80). Father’s odds ratio who was educated at the level of secondary or higher was significant, but lower than mother’s (OR, 1.30; 95% CI, 1.03–1.65).

Greater wealth increased the odds of completing basic immunization in the crude result. After adjusting covariates, only the third level of wealth showed significant odds ratio (OR, 1.52; 95% CI, 1.01–2.27). The source of MCH information had significant impact on the likelihood of full basic immunization. If health professionals provided information about MCH, the likelihood of full basic immunization increased (OR, 1.24; 95% CI, 1.02–1.52); relatives/friends (OR, 1.38; 95% CI, 1.12–1.71) and media (OR, 1.32; 95% CI, 1.08–1.61) as MCH information source did increase likelihood of full basic immunization. The likelihood of full basic immunization was higher (1.61 OR; 95% CI, 1.17–2.22) for those who received three sessions of ANC, and 1.39-fold higher (95% CI, 1.09–1.79) for those who received more than four sessions of ANC. Mother’s age, residence, low-level health workers, MCH information source, and place of delivery were not significant in multiple logistic regression.

## Discussion

In this study, we examined the coverage, timing, and determinants of full immunization among children aged 0–23 months in Sindh, Pakistan, based on survey data. Just over half (51.3%) of all children had received full basic timely immunizations, and the key determinants for full immunization were children’s age, number of living children, parents’ educational level and wealth, MCH information sources, number of ANC sessions, and assistance during delivery.

Since the Expanded Program on Immunization (EPI) started in Pakistan in 1978 with the goal of vaccinating children aged 0–23 months, full basic immunization rate has been increasing. In this study, the average full basic immunization rate in 2013–2014 in Sindh was 51.3%, which was almost double the rate (28%) in 2006–2007 in Sindh [22]. However, the rate was still lower when compared to the rates in other low- and middle-income countries [9, 10, 23] as well as the goal rate of World Health Organization (WHO) and United Nations International Children’s Fund (UNICEF) (at least 90% by 2015) [24].

Children’s age was a significant determinant of full immunization. Interestingly, there was significantly lower immunization coverage in the age category 10–13 weeks. Late immunizations may influence this finding [9, 20]. Late immunizations were frequently reported for Polio and DTP in Pakistan [20] and for BCG and DTP-HepB-Hib (diphtheria, tetanus, whooping cough, hepatitis B, haemophilus influenza type B) in rural Ghana [9]. Median delays for immunization was two-four weeks [9], which explained why the immunization coverage in the age category 14 weeks-8 months old increased after 10–13 weeks of birth. Delays for the first immunization led to delays for second and third Polio and DTP, which indicated that the children remained vulnerable to vaccine-preventable diseases [20]. The common reasons of parents’ vaccine hesitancy, defined as delays in acceptance and/or refusal of vaccination [25], were socioeconomic status, cultural factors, religious reasons, personal beliefs, social/peer environment, philosophical reasons, safety concerns, a desire for more information from healthcare providers, and lack of access to vaccine services [18, 25–29].

WHO recommended that countries achieve vaccination coverage of at least ninety percent nationally and at least eighty percent in every district by 2020 [30]. As we know, the benefits of vaccination are experienced even by unvaccinated children through herd immunity [31]. However, low vaccination coverage (51.3%) and delays for immunization in Pakistan results in the loss of herd immunity which indicates an increased risk of exposure to vaccine-preventable diseases in unvaccinated infants. Previous studies found that there were substantial differences in timeliness of immunization across parents’ educational levels and socioeconomic status [9, 20].

Family characteristics should be considered in determining strategies to improve immunization rates. In this study, the number of living children in the household was associated with successfully completing the basic immunization. More children mean more exposure to the knowledge about immunization and more adherence to the schedule because of repeated learning curve and education effect. Indeed, education level of both mother and father was associated with their children completing basic immunization, which is in line with the findings of previous studies [9, 19, 22, 32]. Parents’ low educational level may influence their general health literacy and lessen their ability to properly understand the benefits of timely and complete immunization and to have better knowledge of vaccine-preventable diseases [22, 33–35].

These findings suggest that educational interventions aimed at less educated parents may have the potential to improve vaccination coverage in Pakistan, where the overall adult literacy rate was 54.9% between 2008 and 2012 [36]. A study was conducted to provide a simple educational intervention (easy to-understand pictorial cards, using very simple language, to convey three key messages) designed for low-literate populations in Pakistan [37]. This intervention improved DPT-3/Hepatitis B vaccine completion rates by 39%. Another study in Malaysia also showed the effect of a short educational intervention (an animated movie and lecture using simple understandable language) on improving parents’ knowledge of immunization [38].

Previous studies showed a positive relationship between residing in wealthier households with complete full immunization status [10, 20, 33, 39] but, wealth was not significantly associated with full basic immunization in this study. One possible reason is that wealth did not have much effect on the full immunization coverage as it used to be because immunization provided by the EPI program is free, and public efforts to access vulnerable mothers and infants are continued. Another possible reason is that Sindh provinces is a more unequal and polarized area than Punjab, Khyber-Pakhtunkhwa, and Balochistan in Pakistan [20, 26] and, therefore, different determinants based on wealth quintiles may influence immunization coverage compared with other study findings.

Having access to health information could play a pivotal role in improving mothers’ awareness regarding full immunization. In this study, mothers who had received information about MCH in the last year–whether from healthcare professionals, other persons, or the media–showed higher odds of completing basic immunization than those who did not. This is supported by previous studies, including in Pakistan, that found contact with health facilities and access to mass media were positively associated with full immunization [10, 16, 22, 23]. Contact with health facilities is a proxy for interactions with healthcare professionals, which provides an opportunity to receive information about immunization [23, 40, 41].

Likewise, access to radio, television, phones, and computers permits women to receive health-related information more easily [42–44]. However, recent studies concerned about the adverse effects of social media on completing basic immunization, because social media often has contributed to a dissemination of rumors, misconceptions or inaccurate beliefs about vaccination that ultimately led to the higher degrees of vaccine hesitancy and lower immunization rates [45–48]. Further studies should verify the accuracy of information provided by radio, television, phones, and the internet and should develop appropriate television/online programs to convey accurate information regarding immunization and to improve the full basic immunization in Pakistan. Previous studies reported that other persons, such as mother-in-law, relatives, and friends, played a significant role as health information source in Pakistan [49, 50]. They helped mother’s birth preparedness and health behaviors, which might lead to increase in full vaccination.

In the current study, making at least three ANC visits was significantly associated with completing basic immunization. Most Pakistani women in Sindh received specific elements of ANC, such as checking blood pressure, urine testing, blood tests, iron supplementation, tetanus immunizations, weight measurement, and counseling about danger signs of pregnancy [17]. Studies conducted in Ethiopia [51] and the Philippines [33] reported that infants whose mothers received the WHO recommended number of four ANC visits were significantly more likely to have their children immunized. In India, the immunization rate of children aged 12 to 23 months among a group of mothers who had one to two ANC visits was 13%, whereas the immunization rate among a group who had more than two visits were 19% [15]. ANC visits may be important signal to show mothers’ ready access to a health facility (i.e., a short distance from a health facility or having transportation options/alternatives). Furthermore, increased contact with the healthcare facility for obtaining ANC would improve the full immunization rate of children because mothers would have more opportunity to be informed about child healthcare, including the importance of full immunization, and to be encouraged by healthcare staff to use the healthcare service [15, 33]. Policy makers should assess the obstacles of receiving ANC in Pakistan based on mother’s background (i.e., socioeconomic status, residence, number of health facilities, cultural norms/beliefs) and develop strategies to increase ANC check-up (i.e., increase in number of health facilities/female healthcare professionals, free public transportation for pregnant women, mass campaigns), which may ultimately result in an increase in the immunization rate.

This cross-sectional study has several limitations. First, because of the cross-sectional design, we could not assess the causal relationship between immunization coverage and any of the other independent variables as they were collected at the same time point. Prospective studies determining predictors of full immunization coverage are needed to provide evidence for developing educational intervention studies. Second, this study was restricted to the mother’s last live birth in the two years prior to the study in one of four provinces in Pakistan; therefore, the findings cannot be generalized to all children in Pakistan. Further studies are needed to include all children under five years of age in all provinces in Pakistan. Lastly, the basic immunization rate was extracted from the immunization cards, which may have led to selection bias because infants whose parents had not immunization cards were excluded. Selection bias may be overcome through case-control matching, in which cases are selected based on the presence of the immunization cards and matched to controls that do not have the immunization cards. The refusal rate among respondents could have introduced response bias.

Despite the limitation, the study findings have important implications for countries who aim to improve immunization coverage and decrease the infant mortality rate. Mass immunization campaigns to stress the benefits of timely and complete immunization are needed, especially targeting parents in low educational and socioeconomic classes. The activities of mass media and healthcare professionals should be reinforced to improve women’s awareness of timely and complete immunization and importance of health care utilization.

## Conclusions

Increasing childhood immunization coverage rates remains a national public health goal in low income countries. The immunization completion rate among children aged 0–23 months in Pakistan has been increased since the EPI program was initiated by the WHO in 1978, but was still lower than the rates of other low- and middle-income countries as well as the goal of the WHO and UNICEF. This study provided strong support for further efforts to improve the full basic immunization rate by identifying the key determinants of complete and timely childhood immunization coverage. Low vaccination coverage and delays for immunization results in the loss of herd immunity which lead to the outbreaks of vaccine-preventable diseases in unvaccinated infants in Pakistan. Policy-makers should identify mothers at risk of low immunization coverage and make the effort to tailor interventions informing mothers of the need for full immunization and motivating them to receive regular WHO-recommended ANC. Further longitudinal studies are needed to explore the factors associated with timely and complete full immunization and to determine the effect of educational interventions and mass immunization campaigns on completing immunizations and on infant mortality rates.

## Supporting information

S1 Dataset (XLSX)

## References

[1] Organization WH. Immunization leaders call for increased political support for immunization in Pakistan World Health Organization2015. Available from: http://www.emro.who.int/media/news/political-support-immunization-pakistan.html.

[2] Statistics PBo. Pakistan Social and Living Standards Measurement Survey (2014–15): Pakistan Bureau of Statistics; 2016 Available from: http://www.pbs.gov.pk/sites/default/files//pslm/publications/PSLM_2014-15_National-Provincial-District_report.pdf.

[3] Fine P, Eames K, Heymann DL. “Herd immunity”: a rough guide. Clin Infect Dis. 2011;52(7):911–6. 10.1093/cid/cir007 21427399

[4] Kroger AT, Atkinson WL, Marcuse EK, Pickering LK. General recommendations on immunization: recommendations of the Advisory Committee on Immunization Practices (ACIP). MMWR Recomm Rep. 2006:1–47. 17136024

[5] Plotkin SA, Plotkin SA. Correlates of vaccine-induced immunity. Clin Infect Dis. 2008;47(3):401–9. 10.1086/589862 18558875

[6] Hosseinpoor AR, Victora CG, Bergen N, Barros AJ, Boerma T. Towards universal health coverage: the role of within-country wealth-related inequality in 28 countries in sub-Saharan Africa. Bull World Health Organ. 2011;89:881–9. 10.2471/BLT.11.087536 22271945 PMC3260894

[7] Omer SB, Salmon DA, Orenstein WA, Dehart MP, Halsey N. Vaccine refusal, mandatory immunization, and the risks of vaccine-preventable diseases. N Engl J Med. 2009;360(19):1981–8. 10.1056/NEJMsa0806477 19420367

[8] National Institute of Population Studies and ICF International. Pakistan Demographic and Health Survey 2012–13. 2014.

[9] Gram L, Soremekun S, ten Asbroek A, Manu A, O'leary M, Hill Z, et al Socio‐economic determinants and inequities in coverage and timeliness of early childhood immunisation in rural G hana. Trop Med Int Health. 2014;19(7):802–11. 10.1111/tmi.12324 24766425

[10] Kawakatsu Y, Tanaka J, Ogawa K, Ogendo K, Honda S. Effects of three interventions and determinants of full vaccination among children aged 12–59 months in Nyanza province, Kenya. Public Health. 2015;129(11):1530–8. 10.1016/j.puhe.2015.07.008 26278475

[11] Rahman M, Obaida-Nasrin S. Factors affecting acceptance of complete immunization coverage of children under five years in rural Bangladesh. Salud Publica Mex. 2010;52(2):134–40. 20485870 10.1590/s0036-36342010000200005

[12] Onsomu EO, Abuya BA, Okech IN, Moore D, Collins-McNeil J. Maternal education and immunization status among children in Kenya. Matern Child Health J. 2015;19(8):1724–33. 10.1007/s10995-015-1686-1 25636652

[13] Fatiregun AA, Okoro AO. Maternal determinants of complete child immunization among children aged 12–23 months in a southern district of Nigeria. Vaccine. 2012;30(4):730–6. 10.1016/j.vaccine.2011.11.082 22137878

[14] Ozcirpici B, Sahinoz S, Ozgur S, Bozkurt A, Sahinoz T, Ceylan A, et al Vaccination coverage in the South-East Anatolian Project (SEAP) region and factors influencing low coverage. Public Health. 2006;120(2):145–54. 10.1016/j.puhe.2005.04.008 16260009

[15] Dixit P, Dwivedi LK, Ram F. Strategies to improve child immunization via antenatal care visits in India: a propensity score matching analysis. PLoS One. 2013;8(6):e66175 10.1371/journal.pone.0066175 23824555 PMC3688852

[16] Douba A, Aka LBN, Yao GHA, Zengbé-Acray P, Akani BC. Sociodemographic factors associated with incomplete immunization of children aged 12 to 59 months in six West African countries. Sante Publique. 2015;27(4):575–84. 26751932

[17] Agha S, Williams E. Maternal and Child Health Program Indicator Survey 2013, Sindh Province. MNCH Services Component, USAID/Pakistan MCH Program Karachi, Pakistan: Jhpiego. 2013.

[18] Agha S, Tappis H. The timing of antenatal care initiation and the content of care in Sindh, Pakistan. BMC Pregnancy Childbirth. 2016;16(1):190 10.1186/s12884-016-0979-8 27460042 PMC4962355

[19] Rainey JJ, Watkins M, Ryman TK, Sandhu P, Bo A, Banerjee K. Reasons related to non-vaccination and under-vaccination of children in low and middle income countries: findings from a systematic review of the published literature, 1999–2009. Vaccine. 2011;29(46):8215–21. 10.1016/j.vaccine.2011.08.096 21893149

[20] Zaidi SMA, Khowaja S, Kumar Dharma V, Khan AJ, Chandir S. Coverage, timeliness, and determinants of immunization completion in Pakistan: evidence from the Demographic and Health Survey (2006–07). Hum Vaccin Immunother. 2014;10(6):1712–20. 10.4161/hv.28621 24784118 PMC5396236

[21] Jolliffe I. Wiley StatsRef: Statistics Reference Online John Wiley & Sons, 2014 10.1002/9781118445112.stat06472

[22] Bugvi AS, Rahat R, Zakar R, Zakar MZ, Fischer F, Nasrullah M, et al Factors associated with non-utilization of child immunization in Pakistan: evidence from the Demographic and Health Survey 2006–07. BMC Public Health. 2014;14(1):232. 24602264 10.1186/1471-2458-14-232 PMC3973983

[23] Russo G, Miglietta A, Pezzotti P, Biguioh RM, Mayaka GB, Sobze MS, et al Vaccine coverage and determinants of incomplete vaccination in children aged 12–23 months in Dschang, West Region, Cameroon: a cross-sectional survey during a polio outbreak. BMC Public Health. 2015;15(1):630. 26156158 10.1186/s12889-015-2000-2 PMC4496879

[24] World Health Organization & United Nations International Children's Emergency Fund. Global immunization vision and strategy, 2006–2015 Geneva, Switzerland and New York, USA: World Health Organization and United Nations International Children' s Emergency Fund: 2005.

[25] MacDonald NE. Vaccine hesitancy: Definition, scope and determinants. Vaccine. 2015;33(34):4161–4. 10.1016/j.vaccine.2015.04.036 25896383

[26] McKee C, Bohannon K. Exploring the reasons behind parental refusal of vaccines. The J Pediatr Pharmacol Ther. 2016;21(2):104–9. 10.5863/1551-6776-21.2.104 27199617 PMC4869767

[27] Dubé E, Gagnon D, Ouakki M, Bettinger JA, Witteman HO, MacDonald S, et al Measuring vaccine acceptance among Canadian parents: A survey of the Canadian Immunization Research Network. Vaccine. 2018;36(4):545–52. 10.1016/j.vaccine.2017.12.005 29233605

[28] Napolitano F, D'Alessandro A, Angelillo IF. Investigating Italian parents' vaccine hesitancy: A cross-sectional survey. Hum Vaccin Immunother. 2018: 14(7):1558–65. 10.1080/21645515.2018.1463943 29641945 PMC6067864

[29] Larson HJ, de Figueiredo A, Xiahong Z, Schulz WS, Verger P, Johnston IG, et al The state of vaccine confidence 2016: global insights through a 67-country survey. EBioMedicine. 2016;12:295–301. 10.1016/j.ebiom.2016.08.042 27658738 PMC5078590

[30] World Health Organization. Immunization coverage—fact sheet World Health Organization. 2017. Available from: http://www.who.int/mediacentre/factsheets/fs378/en/.

[31] Kim TH, Johnstone J, Loeb M. Vaccine herd effect. Scand J Infect Dis. 2011;43(9):683–9. 10.3109/00365548.2011.582247 21604922 PMC3171704

[32] Danis K, Georgakopoulou T, Stavrou T, Laggas D, Panagiotopoulos T. Socioeconomic factors play a more important role in childhood vaccination coverage than parental perceptions: a cross-sectional study in Greece. Vaccine. 2010;28(7):1861–9. 10.1016/j.vaccine.2009.11.078 20006570

[33] Bondy JN, Thind A, Koval JJ, Speechley KN. Identifying the determinants of childhood immunization in the Philippines. Vaccine. 2009;27(1):169–75. 10.1016/j.vaccine.2008.08.042 18789997

[34] Mohamud AN, Feleke A, Worku W, Kifle M, Sharma HR. Immunization coverage of 12–23 months old children and associated factors in Jigjiga District, Somali National Regional State, Ethiopia. BMC Public Health. 2014;14(1):865. 25146502 10.1186/1471-2458-14-865 PMC4158082

[35] Morrone T, Napolitano F, Albano L, Di Giuseppe G. Meningococcal serogroup B vaccine: Knowledge and acceptability among parents in Italy. Hum Vaccin Immunother. 2017;13(8):1921–7. 10.1080/21645515.2017.1313940 28441109 PMC5557232

[36] United Nations International Children's Emergency Fund. Pakistan: Statistics 2013. Available from: https://www.unicef.org/infobycountry/pakistan_pakistan_statistics.html.

[37] Owais A, Hanif B, Siddiqui AR, Agha A, Zaidi AK. Does improving maternal knowledge of vaccines impact infant immunization rates? A community-based randomized-controlled trial in Karachi, Pakistan. BMC Public Health. 2011;11(1):239. 21496343 10.1186/1471-2458-11-239 PMC3094245

[38] Awadh AI, Hassali MA, Al-Lela OQ, Bux SH, Elkalmi RM, Hadi H. Does an educational intervention improve parents’ knowledge about immunization? Experience from Malaysia. BMC Pediatr. 2014;14(1):254. 25284603 10.1186/1471-2431-14-254 PMC4287312

[39] Lakew Y, Bekele A, Biadgilign S. Factors influencing full immunization coverage among 12–23 months of age children in Ethiopia: evidence from the national demographic and health survey in 2011. BMC Public Health. 2015;15(1):728. 26224089 10.1186/s12889-015-2078-6 PMC4520202

[40] D'Alessandro A, Napolitano F, D'Ambrosio A, Angelillo IF. Vaccination knowledge and acceptability among pregnant women in Italy. Hum Vaccin Immunother. 2018; 14(7):1573–9. 10.1080/21645515.2018.1483809 29863958 PMC6067873

[41] Napolitano F, Navaro M, Vezzosi L, Santagati G, Angelillo IF. Primary care pediatricians’ attitudes and practice towards HPV vaccination: A nationwide survey in Italy. PLoS One. 2018;13(3):e0194920 10.1371/journal.pone.0194920 29596515 PMC5875794

[42] Zucco R, Lavano F, Anfosso R, Bianco A, Pileggi C, Pavia M. Internet and social media use for antibiotic-related information seeking: Findings from a survey among adult population in Italy. Int J Med Inform. 2018;111:131–9. 10.1016/j.ijmedinf.2017.12.005 29425624

[43] Ghweeba M, Lindenmeyer A, Shishi S, Abbas M, Waheed A, Amer S. What predicts online health information-seeking behavior among egyptian adults? a cross-sectional study. J Med Internet Res. 2017;19(6):e216 10.2196/jmir.6855 28642216 PMC5500779

[44] Bianco A, Zucco R, Nobile CGA, Pileggi C, Pavia M. Parents seeking health-related information on the Internet: cross-sectional study. J Med Internet Res. 2013;15(9) e204 10.2196/jmir.2752 24047937 PMC3785974

[45] Dubé E, Gagnon D, Ouakki M, Bettinger JA, Guay M, Halperin S, et al Understanding vaccine hesitancy in Canada: Results of a consultation study by the Canadian Immunization Research Network. PLoS One. 2016;11(6):e0156118 10.1371/journal.pone.0156118 27257809 PMC4892544

[46] Dubé E, Gagnon D, Nickels E, Jeram S, Schuster M. Mapping vaccine hesitancy—Country-specific characteristics of a global phenomenon. Vaccine. 2014;32(49):6649–54. 10.1016/j.vaccine.2014.09.039 25280436 PMC5355208

[47] Massimi A, Rosso A, Marzuillo C, Prencipe GP, De Soccio P, Adamo G, et al Childhood vaccinations. Validation of a tool for measuring knowledge, attitudes and vaccine hesitancy in pregnant women. Epidemiol Biostatistics Public Health. 2017;14(4): e12625.1–e12625.5.

[48] Vezzosi L, Santagati G, Angelillo IF. Knowledge, attitudes, and behaviors of parents towards varicella and its vaccination. BMC Infect Dis. 2017;17(1):172 10.1186/s12879-017-2247-6 28241788 PMC5327543

[49] Kim YM, Haq ZU, Soomro J, Sultana Z, Faizunnisa A, Agha S. Case study: effects of a media campaign on breastfeeding behaviours in Sindh Province, Pakistan. World Health Popul. 2015;16(2):39–45. 26860762 10.12927/whp.2016.24494

[50] Ghani U, Crowther S, Kamal Y, Wahab M. The significance of interfamilial relationships on birth preparedness and complication readiness in Pakistan. Women Birth. 2018;pii:S1871-5192(17)30702-3. 10.1016/j.wombi.2018.03.005 29606520

[51] Sullivan M-C, Tegegn A, Tessema F, Galea S, Hadley C. Minding the immunization gap: family characteristics associated with completion rates in rural Ethiopia. J Community Health. 2010;35(1):53–9. 10.1007/s10900-009-9192-2 19847631

